# Integrative multi-omics analysis identifies genetically supported druggable targets and immune cell specificity for myasthenia gravis

**DOI:** 10.1186/s12967-024-04994-2

**Published:** 2024-03-24

**Authors:** Jiao Li, Fei Wang, Zhen Li, Jingjing Feng, Yi Men, Jinming Han, Jiangwei Xia, Chen Zhang, Yilai Han, Teng Chen, Yinan Zhao, Sirui Zhou, Yuwei Da, Guoliang Chai, Junwei Hao

**Affiliations:** 1 Department of Neurology, Xuanwu Hospital, National Center for Neurological Disorders, Capital Medical University, Beijing, 100053 China; 2 Beijing Municipal Geriatric Medical Research Center, Beijing, China; 3 Key Laboratory for Neurodegenerative Diseases of Ministry of Education, Beijing, China; 4 Department of Human Genetics, McGill University, Montréal, QC Canada

**Keywords:** Myasthenia gravis, Actionable druggable genome, Mendelian randomization, Genetic colocalization, Cell-type specificity

## Abstract

**Background:**

Myasthenia gravis (MG) is a chronic autoimmune disorder characterized by fluctuating muscle weakness. Despite the availability of established therapies, the management of MG symptoms remains suboptimal, partially attributed to lack of efficacy or intolerable side-effects. Therefore, new effective drugs are warranted for treatment of MG.

**Methods:**

By employing an analytical framework that combines Mendelian randomization (MR) and colocalization analysis, we estimate the causal effects of blood druggable expression quantitative trait loci (eQTLs) and protein quantitative trait loci (pQTLs) on the susceptibility of MG. We subsequently investigated whether potential genetic effects exhibit cell-type specificity by utilizing genetic colocalization analysis to assess the interplay between immune-cell-specific eQTLs and MG risk.

**Results:**

We identified significant MR results for four genes (*CDC42BPB, CD226, PRSS36, and TNFSF12*) using *cis-*eQTL genetic instruments and three proteins (CTSH, PRSS8, and CPN2) using *cis-*pQTL genetic instruments. Six of these loci demonstrated evidence of colocalization with MG susceptibility (posterior probability > 0.80). We next undertook genetic colocalization to investigate cell-type-specific effects at these loci. Notably, we identified robust evidence of colocalization, with a posterior probability of 0.854, linking CTSH expression in T_H_2 cells and MG risk.

**Conclusions:**

This study provides crucial insights into the genetic and molecular factors associated with MG susceptibility, singling out CTSH as a potential candidate for in-depth investigation and clinical consideration. It additionally sheds light on the immune-cell regulatory mechanisms related to the disease. However, further research is imperative to validate these targets and evaluate their feasibility for drug development.

**Supplementary Information:**

The online version contains supplementary material available at 10.1186/s12967-024-04994-2.

## Background

Myasthenia gravis (MG) is a chronic autoimmune disorder of the neuromuscular junction [1]. The clinical hallmark of MG is muscle weakness associated with fatigability, which can lead to potentially life-threatening exacerbations, such as myasthenic crisis, which affects around 15% of individuals with MG, remains the leading cause of mortality among patients [2, 3]. MG is a rare disease with an annual incidence of roughly 10–29 cases per 1 million people and a prevalence ranging from 100 cases to around 350 cases per 1 million people [2]. Unlike congenital myasthenic syndromes, characterized by mutations in different genes encoding molecules important in the neuromuscular junction cause major changes in function and are inherited in classic mendelian patterns [4], it can be broadly stated that MG is a complex disorder resulting from the interplay of genetic and environmental factors, triggering autoimmune responses [5]. The underlying genetic pathogenesis is evidenced by the high disease concordance among identical twins [6], and associations with genes in the major histocompatibility complex (MHC) locus have been recognized for more than 30 years [7]. Over the past few decades, the emergence of genome-wide association studies (GWAS) has identified multiple susceptibility variants beyond the MHC with MG risk, yielding an estimated heritability of 25.6% [8]. In a recent GWAS encompassing 1,873 MG patients and 36,370 healthy individuals, identified significant associations in the *CHRNA1* and *CHRNB1* genes, as well as confirmed the previous association signals at *PTPN22*, *HLA-DQA1/HLA-B* and *TNFRSF11A* [9]. These discoveries shed light on the intricate genetic landscape of MG and provide valuable insights into its underlying mechanisms.

The goal of MG treatment is to achieve complete remission or minimal manifestation status with minimal side effects and eventually to avoid a myasthenic crisis [10]. Despite the availability of standard therapies, including acetylcholinesterase inhibitors, steroids, steroid-sparing immunosuppressants, and thymectomy, symptoms of MG are unsatisfactorily treated in up to half of individuals over the course of their disease [11]. A significant proportion of patients heavily rely on corticosteroid administration, which can result in severe side effects, including infections, osteoporosis, diabetes, glaucoma, and other complications [11]. Furthermore, some patients exhibit inadequate response to conventional treatment, with approximately 10–20% of MG patients classified as having "refractory" MG, emphasizing the pressing demand for innovative therapeutic solutions. Although there are several novel treatment options for MG, the therapeutic aim of complete remission only be achieved in a subset of patients [12], indicating that new safe and effective immunotherapies are desperately needed.

The conventional process of drug discovery and development is a time-consuming and costly endeavor. The integration of genomics into the drug discovery process has become indispensable, providing a vital avenue for expediting the development of novel therapeutic targets [13]. The combination of molecular quantitative trait locus (molQTL) studies, such as gene expression or protein quantitative trait loci (eQTLs or pQTLs), with genome-wide association (GWAS) data allows for the identification of target genes associated with risk variants through causal inference [14]. One approach is through drug target Mendelian randomization (MR), a statistical genetic methodology that leverages genetic variants as instrumental variables to assess the causal relationship between an exposure (like genetically predicted druggable gene expression or protein levels) and a specific outcome (such as MG risk). This approach employs genetic data to simulate the design of a randomized controlled trial (RCT) without requiring a drug intervention (Additional file 1: Figure S1) [15]. By synergistically amalgamating diverse data sources [16–22] (Additional file 2: Table S1) and employing rigorous MR and colocalization analyses, this study endeavor strives to identify potential repurposing opportunities for MG, delving into their potential implications in MG susceptibility. Subsequently, further investigation was conducted on the MR associations that exhibited statistical significance and provided evidence for colocalization, aiming to identify immune-cell-specific effects.

## Methods

### Identification of actionable druggable genes

The druggable genome was defined as described in Finan et al. as a selection of genes [23], which included 4479 genes and divided into 3 Tiers based on druggability levels: Tier 1 contains genes encoding targets of approved or clinical trial drugs, Tier 2 genes encoding targets with high sequence similarity to Tier 1 proteins or targeted by small drug-like molecules, and Tier 3 contains genes encoding secreted and extracellular proteins, genes belonging to the main druggable gene families, and genes encoding proteins with more restricted similarity to Tier 1 targets. After removing duplicate and non-autosomal genes, 4300 of 4479 druggable genes were retained in the subsequent analysis (Additional file 2: Table S2).

### Selection of eQTL genetic instruments for drug target gene expression

To simulate exposure to the corresponding drugs, we sought publicly accessible eQTL data for gene expression that encode these druggable proteins. Publicly available data from the eQTLGen consortium (https://eqtlgen.org/, n = 31,684) was utilized to identify common (minor allele frequency > 1%) single-nucleotide variants (SNVs) associated with the expression of drug target genes in blood [16]. We retrieved the complete *cis-*eQTL results (distance between SNV and gene is < 1 Mb, FDR < 0.05) and allele frequency data from the consortium. The SNPs that were associated with gene expression (*cis-*eQTL P < 5 × 10^–8^) were selected as genetic instrumental variables (IVs). To obtain independent IVs, we conducted LD clumping using the TwoSampleMR R package with genotype data of Europeans from the 1000 Genomes were used as a reference panel. In a 10 Mb window, if LD values (r^2^) of two or more SNPs were smaller than 0.01, these SNPs were considered independent IVs.

### Deriving pQTL genetic determinants of circulating protein levels

We obtained pQTL data from six large-scale genome-wide proteomic GWAS studies, namely the ARIC study [17], the INTEVAL study [18], the KORA F4 study [19], the IMPROVE study [20], the AGES-Reykjavik study [21], and the Framingham Heart study (FHS) [22]. Each of these studies undertook proteomic profiling using either SomaLogic SomaScans or O-link proximal extension assays. We restricted proposed instrumental variants to *cis-*pQTLs for druggable proteins, used a *P* value threshold of 5 × 10^–8^. For proteins derived from the ARIC study [17], we utilized the sentinel *cis-*pQTL specific to each protein as it was available. Regarding proteins from the other five studies [18–22], we employed lead variants categorized as tier 1 instrumental variants according to Zheng et al [24], which were associated with fewer than five proteins and exhibited no heterogeneity across the studies.

### Immune-cell-type-specific eQTL data

The immune-cell-specific RNA expression and eQTL data were acquired from the Database of Immune Cell Expression (DICE, https://dice-database.org/) [25], which included eQTLs from 15 different immune cell types from 91 healthy subjects. The presented cell types account for over 60% of all circulating mononuclear cells, consisting of three innate immune cell types (Classical monocytes, Non-classical monocytes, NK cells), four adaptive immune cell types that have not encountered cognate antigen in the periphery (Naive B cells, Naive CD4^+^ T cells, Naive CD8^+^ T cells, and Naive T_REG_ T cells), six CD4^+^ memory or more differentiated T cell subsets (T_H_1, T_H_1/17, T_H_17, T_H_2, follicular T_FH_, and Memory T_REG_ cells), and two activated cell types (Activated Naive CD4^+^ cells and Activated Naive CD8^+^ T cells) [25]. We used these datasets specifically for follow-up analyses of genetically predicted effects identified to evaluate cell-type specificity.

### GWAS summary statistics of MG

For the primary analysis, the largest MG GWAS reported by Chia et al. was used in this study. Briefly, Chia et al. performed a large-scale GWAS analysis on MG, which included 1,873 patients and 36,370 controls [9] (https://www.ebi.ac.uk/gwas/, GWAS Catalog ID: GCST90093061). In this study, the diagnosis of MG relied on standard clinical criteria, including characteristic fatigable weakness, supported by electrophysiological and/or pharmacological abnormalities. Notably, the study is limited to anti-acetylcholine receptor antibodies (anti-AChR) positive cases, and individuals testing positive for antibodies to muscle-specific kinase (anti-MuSK) were excluded. We summarized the genome-wide significant loci identified in the MG GWAS conducted by Chia et al. in Additional file 2: Table S3. To explore age-dependent genetic heterogeneity in MG, we utilized summary statistics from early-onset MG (GWAS Catalog ID: GCST90093465; 595 cases vs. 2,718 controls, aged 40 years or younger) and late-onset MG (GWAS Catalog ID: GCST90093466; 1,278 cases vs. 33,652 controls) separately. For external validation, summary statistics were obtained from the UK Biobank (http://www.nealelab.is/uk-biobank) (224 MG cases vs. 417,332 controls) and FinnGen Biobank (https://r10.finngen.fi/pheno/G6_MYASTHENIA) (461 MG cases vs. 408,430 controls). The MG phenotype was identified through questionnaires completed by the participants, and data on MG subtypes were not applicable. Detailed information on various GWAS datasets is provided in Table 1. To ensure data integrity, we removed SNPs with duplicate or missing identification (rsID) from the dataset for subsequent analysis.

**Table 1:** Details of GWAS summary data and LDSC of single-trait heritability

Traits	Original study (year)	ID	Sample size	Number of cases	Number of controls	Ethnicity	Global h^2^ (SE)	λGC
MG	Ruth Chia et al. (2022)	GCST90093061	38,243	1,873	36,370	European (U.S., Italy)	0.076 (0.015)	1.059
Early-onset MG		GCST90093465	3,313	595	2,718	European (U.S., Italy)	0.365 (0.135)	1.002
Late-onset LOMG		GCST90093466	34,930	1,278	33,652	European (U.S., Italy)	0.078 (0.015)	1.044
MG	–	phecode-358.1	324,308	224	324,074	European (U.K.)	0.002 (0.001)	1.011
MG	–	finngen_R10_G6	408,891	461	408,430	European (Finnish)	0.001 (0.001)	1.013

*LDSC* Linkage disequilibrium score regression, *ID* GWAS Catalog/UK Biobank/FinnGen ID, *Global h*^*2*^ the estimated SNP-heritability, *SE* standard error, *λ*_*GC*_ genomic inflation factor lambda

### SNP-based heritability calculation

We used Linkage Disequilibrium Score (LDSC) Regression (https://github.com/bulik/ldsc) to estimate the SNP-based heritability (h^2^) of each of the trait, representing the proportion of phenotypic variance that is explained by all common genetic variants included in the analysis. Also, we used single-trait LDSC to estimate genomic inflation factors λGC, used to evaluate the polygenicity and confounding because of population stratification or cryptic relatedness. To minimize bias caused by low interpolation quality, we restricted this analysis to haplotype map 3 SNPs.

### Mendelian randomization analysis

The two-sample MR approach was based on the following assumptions: (i) the genetic variants used as an instrumental variable are associated with target exposure, i.e. gene expression levels and protein levels; (ii) there are no unmeasured confounders of the associations between genetic variants and outcome; (iii) the genetic variants are associated with the outcome only through changes in the exposure, i.e. no pleiotropy. We therefore used a curated genotype–phenotype database (PhenoScanner) [26] to search for associations between variants used to instrument each drug target and other traits that may represent pleiotropic pathways. We used fixed-effects, inverse-variance-weighted MR for proposed instruments that contain more than one variant, and Wald ratio for proposed instruments with one variant. Steiger filtering was applied to exclude variants that were potentially influenced by reverse causation [27]. For proposed instruments with multiple variants, we assessed the heterogeneity across variant-level MR estimates using the Cochrane Q method (mr_heterogeneity option in TwoSampleMR package). Benjamini–Hochberg false discovery rate (FDR) of 0.05 was applied to select best MR estimates with robust signals. The findings of our MR studies are presented as MR estimates (β) or odds ratio (OR) and 95% confidence interval (CI) for the risk of MG per genetically predicted 1-standard deviation (SD) increase in blood gene expression or circulating protein level. We conducted MR analyses using the TwoSampleMR (https://mrcieu.github.io/TwoSampleMR/) package.

### Colocalization analysis

While MR largely mitigates bias from confounding, linkage disequilibrium (LD) between SNPs might be an important source of noncausal associations. For statistically significant MR results, *coloc* (https://github.com/cran/coloc) was used to evaluate the probability of QTL and MG loci sharing a single causal variant [28], which assess potential confounding by LD. To determine the posterior probability of each genomic locus containing a single variant affecting both the gene/protein and the MG risk, we analyzed all SNPs within 1 Mb of the *cis-*eQTL/*cis-*pQTL. Moreover, we evaluated whether each genomic locus harbored a causal variant that influenced both disease risk and the variability in gene expression across the 15 cell-type-specific datasets individually. Assuming a solitary causal variant, four hypotheses can be outlined: H0, proposing the lack of causal variants for both traits; H1, positing the existence of a causal variant for trait 1; H2, suggesting a causal variant for trait 2; H3, postulating two distinct causal variants for traits 1 and 2; and H4, proposing a shared causal variant between the two traits. Statistically significant MR hits with posterior probability for hypothesis 4 (PPH4) > 0.8 (the probability of a shared causal variant) were investigated. Visualization of colocalization results was performed using the *LocusCompareR* R package [29].

## Results

### Overall analysis plan

**Fig. 1:**
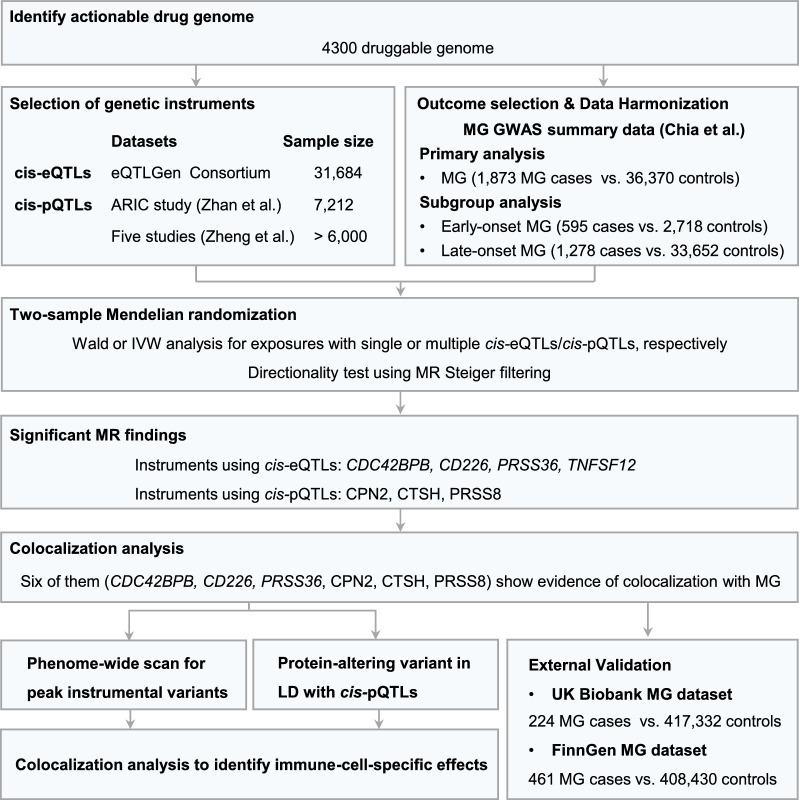
Flow diagram of study design. Using a variety of data sources, this study examined the instruments proposed for actionable druggable proteins, specifically *cis-*pQTL and *cis-*eQTL, against MG GWAS summary statistics. Subsequently, further investigation was conducted on the MR associations that exhibited statistical significance and provided evidence for colocalization, aiming to identify immune-cell-specific effects

The study design is illustrated in Fig. 1. Initially, we identified druggable proteins as described in Finan et al. [23]. These proteins include targets of approved and clinical-phase drugs, proteins resembling approved drug targets and proteins accessible to monoclonal antibodies or drug-like small molecules in vivo. Next, we selected independent genetic variants that act locally on gene expression from eQTLGen Consortium or specifically influence plasma levels of the proteins from six large proteomic GWASs of individuals of European ancestry. The primary analysis involved retrieving summary statistics from the largest MG GWAS dataset of European ancestry [9]. In addition, we conducted a subgroup analysis using GWAS summaries for early-onset MG and late-onset MG separately. Employing the single-trait LDSC method, the SNP heritability for MG was estimated at 0.075 (SE = 0.015), while early-onset MG showed a heritability of 0.3649 (SE = 0.135), and late-onset MG exhibited a heritability of 0.078 (SE = 0.015) (Table 1). Then, genetic colocalization was conducted on MR results that surpassed our significance threshold after accounting for multiple testing, to mitigate potential confounding by LD. Afterward, we performed external replication using MG GWAS summary statistics from the FinnGen Consortium and UK Biobank. Subsequently, we explored whether putative genetic effects may be cell type specific by employing genetic colocalization to assess the interplay between immune-cell-specific eQTLs and MG risk.

### MR analysis with blood gene expression and MG outcome

**Fig. 2:**
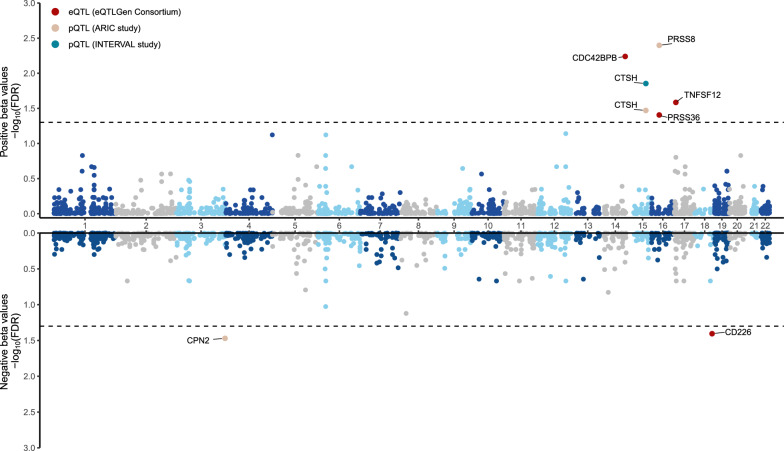
Miami plot with circles representing the MR results for gene/protein on MG. FDR,* P* value (FDR adjust); Black dashed line indicates the threshold for significance (FDR < 0.05) threshold. The x axis is the chromosome and gene start position of each MR finding in the *cis* region. The y axis represents the − log_10_ FDR of the MR findings. MR findings with positive effects (increased level of gene expression/protein associated with increasing the MG risk) are represented by filled circles on the top half of the plot; Conversely, MR findings suggesting a negative effect, implying a reduced level of gene expression or protein linked to an elevated risk of MG, are illustrated in the lower half of the plot

We used two-sample MR to systematically evaluate the evidence for the causal effects of druggable gene expression on MG outcome. Using *cis-*eQTLs as proposed instruments available from the eQTLGen Consortium, four genes (*CDC42BPB*, *TNFSF12*, *CD226* and *PRSS36*) showed significant MR results (Table 2, Fig. 2). Specifically, we found that a 1 standard deviation (SD) increased blood expression of *CDC42BPB* (OR = 1.694; 95% CI 1.361–2.108; *P* = 2.347 × 10^–6^), *TNFSF12* (OR = 1.433; 95% CI 1.214–1.691; *P* = 2.119 × 10^–5^), and *PRSS36* (OR = 3.186; 95% CI 1.805–5.624;* P* = 6.401 × 10^–5^) were significantly associated with increased MG susceptibility, whereas increased *CD226* gene expression (OR = 0.652; 95% CI 0.528–0.804; *P* = 6.205 × 10^–5^) in blood were associated with decreased MG risk (*cis-*eQTL instruments in Additional file 2: Table S4, full MR results in Additional file 2: Table S5, MR scatter plot shown in Additional file 1: Figure S2). We employed Cochran's Q test to assess potential heterogeneity in the IVW results, estimating MR effects across each eQTL and none of the MR signals exhibited evidence of significant heterogeneity. In the replication phase, our study employed GWAS summary data from the UK and FinnGen Biobank datasets. Although MR analysis did not identify any genetically predicted gene expression causally linked to MG risk after FDR correction (full MR results in Additional file 2: Table S5), we observed similar pattern for *CD226* expression with MG susceptibility (OR = 0.595; 95% CI 0.403–0.877; *P* = 8.754 × 10^–03^) (Additional file 1: Figure S3).

**Table 2:** MR-identified genes/proteins with MG risk

Study/Dataset	Genes/Proteins	Method	SNP	Beta	SE	*P* value	FDR	OR (95%CI)	*P*_het_	Colocalization
eQTLGen	CDC42BPB	IVW	rs10143668 rs11627044 rs12435483 rs192018318 rs2403110 rs75817380 rs77630549 rs8015723	0.527	0.112	2.35 × 10^–6^	0.006	1.694 (1.361–2.108)	0.648	0.926
eQTLGen	TNFSF12	IVW	rs10468481 rs11078677 rs12449427 rs1641548 rs2292067 rs60370790 rs62059711 rs72842805 rs78378222	0.36	0.085	2.12 × 10^–5^	0.026	1.433 (1.214–1.691)	0.468	0.305
eQTLGen	CD226	IVW	rs17082031 rs1790974 rs3744856	− 0.428	0.107	6.21 × 10^–5^	0.039	0.652 (0.528–0.804)	0.649	0.815
eQTLGen	PRSS36	Wald ratio	rs78924645	1.159	0.29	6.40 × 10^–5^	0.039	3.186 (1.805–5.624)	NA	0.856
ARIC study	PRSS8	Wald ratio	rs1060506	1.447	0.319	5.69 × 10^–6^	0.004	4.252 (2.275–7.945)	NA	0.982
ARIC study	CPN2	Wald ratio	rs11711157	− 0.2	0.053	1.44 × 10^–4^	0.034	0.819 (0.739–0.908)	NA	0.926
ARIC study	CTSH	Wald ratio	rs2289702	0.197	0.052	1.44 × 10^–4^	0.034	1.218 (1.101–1.348)	NA	0.894
INTEVAL study	CTSH	Wald ratio	rs34593439	0.229	0.057	5.49 × 10^–5^	0.014	1.257 (1.125–1.405)	NA	0.894

The outcome is MG GWAS from Chia et al. (1,873 patients and 36,370 controls); *IVW* inverse-variance weighted, *SE* standard error, *FDR* FDR corrected *P* value, *P*_het_ refers to the heterogeneity calculated using the Cochrane Q method; Colocalization indicates PPH4 between eQTLs/pQTLs and MG GWAS

**Fig. 3:**
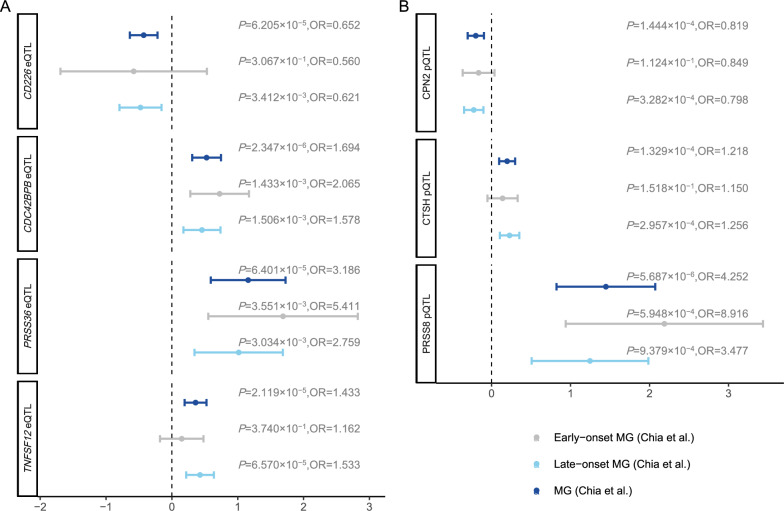
Forest plot showing MR estimate for genetically proxied gene and protein expression on MG and its subgroups. Forest plot showing MR estimate (95% CI) from two sample MR analyses. *P* are unadjusted. *CI* confidence interval. MR estimates of significant MR results used *cis-*eQTL instruments on MG and its subgroups. MR estimates of significant MR results used *cis-*pQTL instruments on MG and its subgroups

In the subgroup analysis discerning early-onset and late-onset MG, coherent patterns emerged (Fig. 3), For instance, a 1 SD increase in genetically predicted *CDC42BPB* expression levels was associated with elevated risk for both early-onset MG (OR = 2.065; 95% CI 1.322–3.225; *P* = 1.433 × 10^–3^) and late-onset MG (OR = 1.578; 95% CI 1.191–2.092; *P* = 1.506 × 10^–3^) risk. Furthermore, pertaining to *CD226* and *TNFSF12*, subgroup MR findings revealed a relationship between *CD226* levels and late-onset MG (OR = 0.621; 95% CI 0.451–0.854; *P* = 3.412 × 10^–3^), as well as an association between *TNFSF12* levels and late-onset MG (OR = 1.533; 95% CI 1.243–1.891; *P* = 6.570 × 10^–5^), while no statistically significant MR estimates were observed in the context of early-onset MG.

**Fig. 4:**
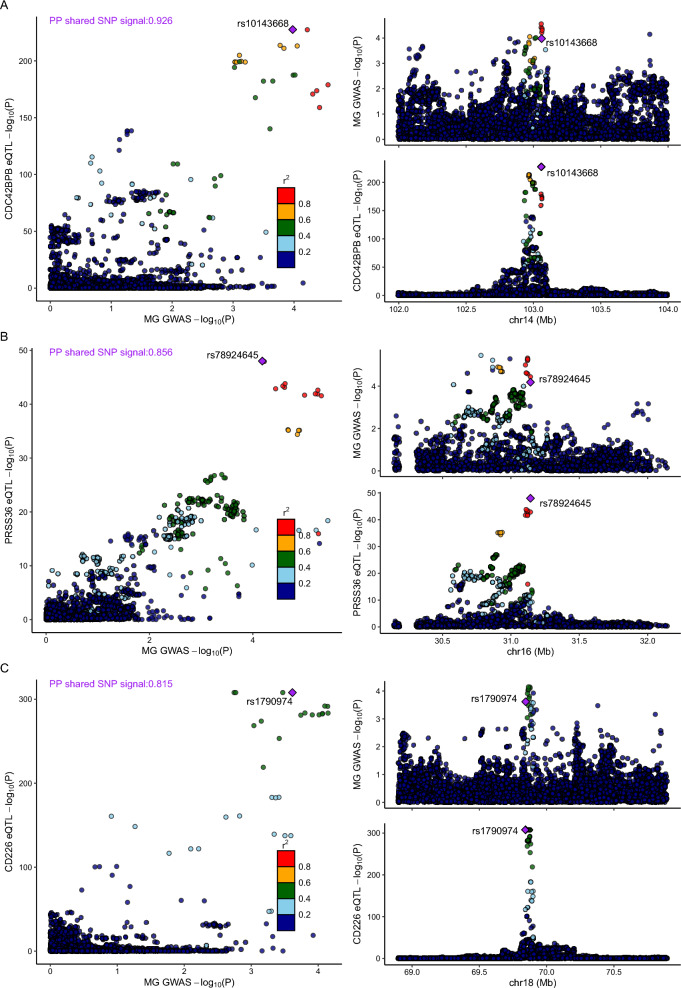
LocusCompare plot depicting colocalization of the top SNP associated with eQTL surrounding CDC42BPB (**A**), PRSS36 (**B**) and CD226 (**C**) and MG GWAS. The top right plots show the association results in the MG GWAS; the bottom right plots represent the corresponding eQTL results; the left plot shows the colocalization of genetic association and eQTL signals. The SNP indicated by the purple diamond is the SNP for which the European LD information is shown

We then performed genetic colocalization analyses to evaluate the probability of shared single causal variants between MG loci and eQTLs. Three loci (*CDC42BPB*, *CD226*, and *PRSS36*) exhibited profiles suggesting causal relationships, except for *TNFSF12* (Fig. 4, Additional file 2: Table S6). Furthermore, we investigated the associations between index *cis-*eQTLs (specifically, rs1790974 for *CD226* levels, rs10143668 for *CDC42BPB* levels, and rs78924645 for *PRSS36* levels) and more than 5,000 other diseases, traits or proteins levels as documented in PhenoScanner [26] (Additional file 1: Figure S4), which suggest potential horizontal pleiotropic effects in our MR analysis.

### MR analysis of circulating proteome identifies actionable targets in MG

**Fig. 5:**
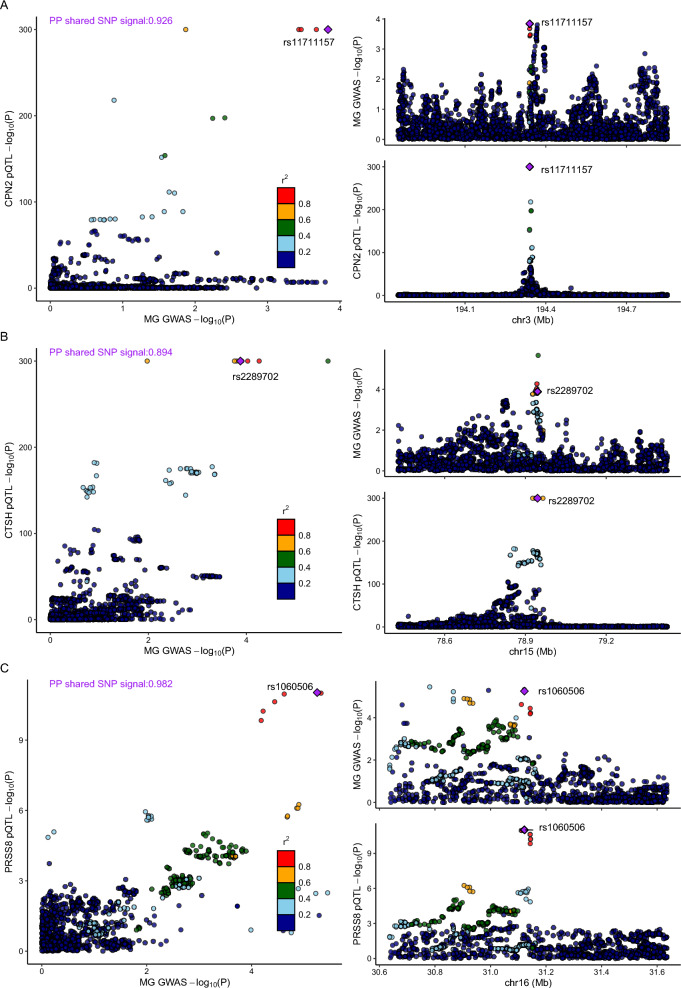
LocusCompare plot depicting colocalization of the top SNP associated with pQTL surrounding CPN2 (**A**), CTSH (**B**) and PRSS8 (**C**)and MG GWAS. The top right plots show the association results in the MG GWAS; the bottom right plots represent the corresponding pQTL results; the left plot shows the colocalization of genetic association and eQTL signals. The SNP indicated by the purple diamond is the SNP for which the European LD information is shown

Using proposed *cis-*pQTL instruments, MR analyses revealed significant associations between the levels of three circulating proteins (PRSS8, CTSH, CPN2) and MG susceptibility in the primary analysis, after FDR correction (*cis-*pQTL instruments in Additional file 2: Table S7, and full MR results in Additional file 2: Table S8), which exhibit compelling evidence of colocalization with MG risk (Fig. 5, Additional file 2: Table S9). Notably, among these proteins, circulating CTSH exhibited associations that were replicated in two independent proteomic studies (Fig. 2, Table 2). The estimated associations with MG per 1 SD increase in the genetically predicted circulating CTSH level were consistent between the INTERVAL (OR = 1.257; 95% CI 1.125–1.405; *P* = 5.49 × 10^–4^) and ARIC (OR = 1.218; 95% CI 1.101–1.348; *P* = 1.330 × 10^–4^) studies and displayed strong evidence of colocalization (Fig. 5). Despite the MR analysis failing to identify any genetically predicted circulating protein levels associated with MG susceptibility using the proposed *cis-*pQTL instruments and GWAS summary data from the FinnGen and UK Biobank datasets after FDR correction, a persistent association for Cathepsin H (CTSH) abundance abundance with MG was evident. This sustained association suggests an elevated risk of MG across three MG outcomes. A SD increase in genetically predicted circulating CTSH abundance was found to be associated with an elevated risk of MG (minimum OR = 1.218; 95% CI 1.101–1.348; *P* = 1.329 × 10^–4^), and this trend was consistently observed in both the UK Biobank (OR = 1.275; 95% CI 1.003–1.620; *P* = 4.713 × 10^–2^) and FinnGen Biobank (OR = 1.185; 95% CI 1.017–1.381; *P* = 2.942 × 10^–2^) datasets (Additional file 1: Figure S3).

Additionally, subgroup analyses can provide further insights into the nuanced relationships between these proteins and MG susceptibility (Fig. 3). Genetically predicted circulating CTSH abundance was associated with an increased risk of late-onset MG (OR = 1.256; 95% CI 1.110–1.421; *P* = 2.957 × 10^–4^). Also, genetically predicted elevation in circulating PRSS8 abundance is associated with an increased risk of MG (OR = 4.252; 95% CI 2.275–7.945; *P* = 5.69 × 10^–6^), as well as heightened risks for both early-onset MG (OR = 8.916; 95% CI 2.558–31.083; *P* = 5.948 × 10^–6^) and late-onset MG (OR = 3.477; 95% CI 1.662–7.275; p = 9.379 × 10^–4^).Conversely, a genetically proxied increase in circulating CPN2 levels demonstrated an association with decreased MG risk (OR = 0.819; 95% CI 0.739–0.908; *P* = 1.444 × 10^−4^) and also late-onset MG (OR = 0.798; 95% CI 0.706–0.903; *P* = 3.282 × 10–^04^). Due to the limited number of *cis-*acting pQTLs available for each protein, applying classic sensitivity methods to test MR assumptions becomes challenging.

We also searched in the PhenoScanner database to assess potentially pleiotropic effects of the cis-pQTLs of MR-prioritized proteins by testing the association of cis-pQTLs with other diseases or traits [26] (Additional file 1: Figure S4). We observed no evidence of pleiotropic effects for the cis-pQTL associated with CPN2 (rs11711157). We discovered an association between the CTSH *cis-*pQTL (rs34593439) and narcolepsy, a condition for which immune-mediated dysregulation has been considered as one of the potential causes [30]. Also, we found that PRSS8 *cis-*pQTL (rs1060506) was associated with a broad spectrum of weight-related traits, including whole body fat mass and body fat percentage, among others, which was consistent with the findings for *cis-*eQTL for *PRSS36*. This suggests that our MR estimate of the effect of the above-mentioned proteins on MG susceptibility may have been biased by the stated confounders.

### Identifying immune-cell-specific effects

**Fig. 6:**
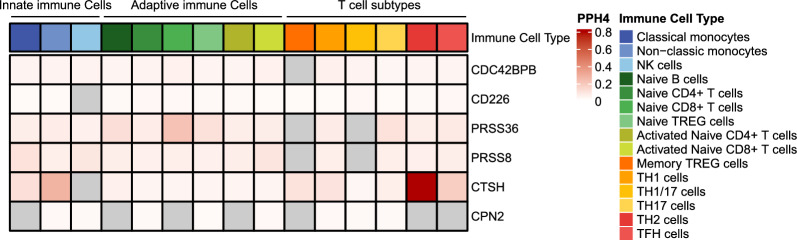
Colocalization results between GWAS and eQTLs across immune cell types. PPH4 of shared genetic signal between GWAS and eQTLs for MG across different immune cell types

Given the pivotal role of immune cells in the pathogenesis of MG, we aim to investigate whether potential genetic effects exhibit specificity toward certain cell types. Leveraging immune cell eQTL datasets from the DICE database, we conducted a comprehensive genetic colocalization analysis for each of the six identified loci (*CDC42BPB*, *CD226*, *PRSS36*, *PRSS8*, *CTSH*, and *CPN2*) from the preceding analysis. Remarkably, we identified robust evidence of colocalization, with a posterior probability of 0.854, linking *CTSH* expression in T_H_2 cells and MG risk (Additional file 2: Table S10, Additional file 1: Figure S5). T_H_2 cells are renowned for their capacity to produce cytokines that stimulate B cell activation and differentiation of B cells, essential for antibody production [31]. Furthermore, while analyzing data from other immune cell subsets, we observed less pronounced evidence specific to individual cell types (Fig. 6). The identification of a colocalization signal within a cell type relevant to MG pathogenesis suggests a potential direct contribution of the genetic variant to disease development by influencing gene expression within that specific cell subset.

## Discussion

Gaining an in-depth understanding of the intricate relationship between genetic discoveries and pharmaceutical targets holds paramount importance in effectively translating GWAS findings into clinical applications [32]. Our study assumes pioneering significance as the first to center on actionable druggable genes, unlocking the potential of drug repurposing strategies within the domain of MG treatment. Notably, we identified three genes (*CDC42BPB*, *CD226*, and *PRSS36*) and three proteins (PRSS8, CTSH, and CPN2) as having significant MR results using cis-eQTL and cis-pQTL genetic instruments, which also exhibit compelling evidence of colocalization with MG. Additionally, through a meticulous exploration of immune cell-specific effects, this study may shed light on mechanistic insights underlying the loci associated with MG.

Our MR analysis establishes a compelling link between genetic variants associated with increased *CDC42BPB* gene expression and an elevated MG susceptibility. This finding underscores the potential of *CDC42BPB* inhibitors as a promising avenue for therapeutic intervention. *CDC42BPB* is a serine/threonine protein kinase intricately involved in regulating actin cytoskeleton dynamics and cell contraction. Several small molecules or biological inhibitors of *CDC42BPB*, such as SR-7826, BDP8900 and BDP9066 [33], have exhibited antitumor activity. However, a comprehensive understanding of the mechanistic underpinnings through which elevated *CDC42BPB* contributes to the pathophysiology of MG necessitates further investigation. Conversely, we observed a protective effect associated with increased *CD226* gene expression against MG. *CD226*, also known as DNAX accessory molecule-1 (DNAM-1), is a member of the immunoglobulin superfamily and is expressed on various immune cells, including natural killer (NK) and T cells [34]. *CD226*, along with the inhibitory receptors TIGIT and CD96, constitutes cell-surface receptor family 3, binding to nectin and nectin-like proteins [35]. Prior research emphasizes the potent roles of this receptor family in regulating tumor immunity [36]. These findings underscore the importance of maintaining the expression of the activating receptor CD226, in orchestrating effective immune responses. Genetic polymorphisms within the *CD226* gene have been associated with serval autoimmune diseases, including multiple sclerosis [37]. Building on these insights, we hypothesize that targeted manipulation of *CDC42BPB* and *CD226* expression could potentially serve as a potent therapeutic strategy for managing MG. Nonetheless, a comprehensive validation of these proposed therapeutic targets necessitates further rigorous investigation.

Our study emphasizes a consistent and robust association between genetically predicted circulating Cathepsin H abundance and an elevated risk of MG across diverse datasets, including the UK Biobank and FinnGen Biobank. The subgroup analyses further revealed nuanced insights, particularly the association between genetically predicted circulating CTSH abundance and an increased risk of late-onset MG. Additionally, the posterior probability that CTSH expression levels in T_H_2 cells and MG outcomes shared a single causal signal in the 1-Mb locus around the cis-pQTL, rs12148472, was 0.8854 for MG susceptibility (Additional file 1: Figure S5). This observation implies that CTSH abundance might serve as a biomarker or contribute to the biological mechanisms underlying MG. *CTSH* encodes cathepsin H, a member of the papain-like cysteine proteases that are involved in major histocompatibility complex class II antigen presentation. Notably, *CTSH* has garnered attention for its involvement in type 1 diabetes [38] and narcolepsy risk [39], both of which prominently feature autoimmune components. Worth noting is the fact that other members of the cathepsin family, such as cathepsin S and cathepsin K, have received more attention as drug targets for autoimmune diseases. For example, cathepsin S inhibitors have been investigated for their potential in treating diseases like and multiple sclerosis [40].

Furthermore, our study provided robust genetic evidence supporting a potential causal role of increased CPN2 protein levels in reducing the risk of MG. CPN2, also known as Carboxypeptidase N Subunit 2, forms a complex with enzymatically active small subunits (CPN1). CPN plays a pivotal role as a zinc metalloprotease responsible for cleaving and partially inactivating anaphylactic peptides, specifically complement component 3a (C3a) and C5a, within the classical and lectin pathways of complement activation [41]. Protein–protein interaction studies using the STRING database (https://string-db.org/) have shown interactions between CPN2 and C5 (Additional file 1: Figure S6). It is widely recognized that dysregulated complement activation is a primary pathogenic mechanism in MG. Notably, C5 inhibitors, such as Eculizumab, have proven effective in preventing complement-dependent membrane attacks at the neuromuscular junction, presenting a promising avenue for the treatment of MG [42]. Future research is warranted to investigate the potential therapeutic implications of CPN2 in MG.

A significant hurdle in our analytical pursuit lay in discerning whether the association with MG risk stemmed from PRSS36, PRSS8, or both entities, given the substantial correlation between the *cis-*eQTL instrument for *PRSS36* (rs78924645) and *cis-*pQTL instrument (rs1060506) for PRSS8 (r^2^ = 0.93 in 1000 Genomes Project European ancestry participants). Considering that both PRSS36 and PRSS8 belong to the serine protease family, a group of proteolytic enzymes involved in diverse biological processes, we regard them as potential drug targets for MG. It is worth noting that PRSS8 inhibits TLR4-mediated inflammation in human and mouse models of inflammatory bowel disease [43], thereby implicating its plausible relevance to TLR4-mediated inflammation pathways in the context of MG. As such, the identification of *PRSS36* and PRSS8 as putative targets holds promise; however, the trajectory towards their validation as credible therapeutic avenues warrants further inquiry, coupled with a comprehensive assessment of their viability for subsequent drug development endeavors.

Genetic variation controls transcriptional regulation in a cell type-specific manner to regulate immune pathways [44, 45]. The cell-type specificity of the effects elicited by common genetic variants on both gene and protein expression engenders a pronounced reliance on the precise cellular contexts. In navigating this intricacy, the relationships underpinning eQTL and pQTL manifest a profound dependence on the distinct cell types under scrutiny. In this study, we embarked on a comprehensive exploration, utilizing genetic colocalization analysis, to delineate the interplay between immune-cell-specific eQTLs and MG susceptibility, thereby unearthing the underlying molecular mechanisms. This approach yielded valuable insights into the genetic orchestration governing CTSH expression across an array of distinct cell types. Of note is the robust colocalization unveiled between CTSH expression within T_H_2 cells and the propensity for MG risk, signifying a putative involvement of CTSH expression within this cellular subtype in driving the genesis and advancement of MG. This revelatory aspect of our study underscores the imperative of acknowledging and integrating cell type heterogeneity within the realm of future research pursuits aimed at identifying potential therapeutic targets for MG. This newfound awareness stands poised to wield transformative influence in guiding the trajectory of future scientific explorations and therapeutic innovations targeting MG and other akin disorders.

The current study notably complements and extends previous efforts by employing key approaches to protect against potential biases, strengthen causal inference and enhance understanding of potential mechanisms. With our rigorous instrument selection process that used comprehensive datasets on gene expression and plasma protein levels, we have facilitated a thorough evaluation of actionable drug targets, notably including the previously unexplored CTSH. Our study, enriched with multi-omics data, has successfully overlooked targets that do not align with the potentially druggable gene targets prioritized in the Chia et al. study, as indicated by the Priority Index analysis [9, 46]. It is crucial to underscore that the peak *cis-*eQTL or *cis-*pQTL identified at each locus did not attain the genome-wide significance level threshold. Consequently, these loci were not deemed significantly associated with MG in Chia et al.'s study. This constitutes a noteworthy extension to previous study, emphasizing our pivotal role in contributing novel insights that surpass the confines of earlier investigations.

Several limitations need to be considered in our study. Firstly, only a small proportion of genes/proteins may be effectively instrumented by multiple SNPs, with the majority being instrumented by only two or one SNP. This limitation restricts our ability to conduct sensitivity analyses. Secondly, there is a potential concern related to the presence of epitope-binding artifacts caused by coding variants. These artifacts may introduce spurious signals, potentially leading to false-positive cis-pQTLs. Thirdly, our study is confined to AchR-positive MG cases, so caution is advised when extrapolating our results to patients with anti-MuSK and other autoantibodies. Additionally, although the GWAS summary data integrated into our study already constitute the most extensive MG GWAS dataset currently available, the limited number of cases in both the primary and replication datasets constrained our ability to replicate the MR estimates observed in the discovery cohort. Therefore, it is crucial to conduct further research with larger and more diverse populations, especially including non-European individuals and patients with anti-MuSK and other autoantibodies.

## Conclusions

Overall, this study contributes valuable insights into the genetic and molecular factors associated with MG susceptibility, with CTSH emerging as a potential candidate for further investigation and clinical consideration. Moreover, by laying bare the intricacies of cell-specific genetic influences on gene and protein expression, our investigation serves as a clarion call for a nuanced consideration of cellular contexts in the quest for unraveling the underlying mechanisms of complex diseases like MG. It is imperative to underscore, however, that a requisite next step involves undertaking further studies, encompassing rigorous functional analyses, to definitively validate the viability of these targets and to ascertain their appropriateness for subsequent drug development endeavors.

### Supplementary Information

**Additional file 1: Figure S1.** Mendelian Randomization (MR) vs. Randomized controlled trial (RCT). **Figure S2.** Scatter plots of significant results from genetically predicted gene expression on MG risk in the primary analysis. **Figure S3.** Forest plot showing MR estimate for genetically proxied gene and protein expression on MG outcome across different datasets. **Figure S4.** PhenoScanner disease/trait annotation of the index eQTL/pQTL instrument with other traits. **Figure S5.** LocusCompare plot depicting colocalization of the top SNP associated with eQTL surrounding CTSH in T_H_2 cell and MG GWAS. **Figure S6.** Protein–protein interaction of CPN2 using the STRING database (https://string-db.org/).

**Additional file 2: Table S1.** Basic Characteristics of eQTL Databases, pQTL Studies, and GWAS Datasets in the study. **Table S2.** An overview of druggable proteins and the coverage of genes/proteins in eQTLGen and pQTL studies. **Table S3.** Genome-wide significant loci in MG GWAS from Chia et al. **Table S4.** The *cis-*eQTLs instruments used for drug target gene expression on MG risk in the primary analysis. The outcome is MG GWAS from Chia et al. (1,873 patients and 36,370 controls). **Table S5.** MR full results using *cis-*eQTLs on MG risk across different datasets. **Table S6.** Colocalization results using cis-eQTLs on MG across different datasets. **Table S7.** The *cis-*pQTLs instruments used for protein expression on MG risk in the primary analysis. The outcome is MG GWAS from Chia et al. (1,873 patients and 36,370 controls). **Table S8.** MR full results of proteins using sentinel *cis-*pQTLs from six large proteomic studies on MG risk across different datasets. **Table S9.** Colocalization results using *cis-*pQTLs on MG risk across different datasets. **Table S10.** Colocalization results for genes/proteins using eQTL from the DICE database.

## Data Availability

The eQTL data from eQTLGen consortium were obtained from https://www.eqtlgen.org/. The eQTL data from DICE were obtained from https://dice-database.org/. The ARIC pQTL summary statistics used in the paper are freely downloaded from ARIC website (http://nilanjanchatterjeelab.org/pwas/). Summary statistics for the MG GWAS are publicly available for download from the GWAS catalog. Data processing was completed using R software (version 3.6.3), with packages TwoSampleMR (version 0.5.4), coloc (version 4.0.4), dplyr (version 1.0.0), readr (version 1.3.1), tidyverse (version 1.3.0), forestplot (version 1.9), plyr (version 1.8.6), devtools (version 2.3.0), remotes (version 2.1.1).
