# Supplementary material for: Integrative multi-omics analysis identifies genetically supported druggable targets and immune cell specificity for myasthenia gravis
Source: J Transl Med. 2024 Mar 24;22:302. doi: 10.1186/s12967-024-04994-2 (PMC10960998; doi:10.1186/s12967-024-04994-2)
Supplement: Supplementary file 1 — Additional file 1: Figure S1. Mendelian Randomization (MR) vs. Randomized controlled trial (RCT). Figure S2. Scatter plots of significant results from genetically predicted gene expression on MG risk in the primary analysis. Figure S3. Forest plot showing MR estimate for genetically proxied gene and protein expression on MG outcome across different datasets. Figure S4. PhenoScanner disease/trait annotation of the index eQTL/pQTL instrument with other traits. Figure S5. LocusCompare plot depicting colocalization of the top SNP associated with eQTL surrounding CTSH in TH2 cell and MG GWAS. Figure S6. Protein–protein interaction of CPN2 using the STRING database (https://string-db.org/). [file 12967_2024_4994_MOESM1_ESM.docx]

Additional material

# Integrative multi-omics analysis identifies genetically supported druggable targets and immune cell specificity for myasthenia gravis

Jiao Li, MD, PhD^1,2,3 #^, Fei Wang, PhD^1,3 #^, Zhen Li, MD^1^, Jingjing Feng, PhD^1^, Yi Men, MD^1^, Jinming Han, MD, PhD^1^, Jiangwei Xia, PhD^1^, Chen Zhang, MD, PhD^1^, Yilai Han, MSc^1^, Teng Chen, PhD^1^, Yinan Zhao, MD, PhD^1^, Sirui Zhou, PhD ^4^, Yuwei Da, MD, PhD^1^, Guoliang Chai, PhD^1,2 *^, Junwei Hao, MD, PhD^1,2,3 *^

^1^ Department of Neurology, Xuanwu Hospital, National Center for Neurological Disorders, Capital Medical University, Beijing, 100053, China.

^2^ Beijing Municipal Geriatric Medical Research Center, Beijing, China.

^3^ Key Laboratory for Neurodegenerative Diseases of Ministry of Education, Beijing, China.

^4^ Department of Human Genetics, McGill University, Montréal, Quebec, Canada.

# Additional file 1

## Figure S1. Mendelian Randomization (MR) vs. Randomized controlled trial (RCT)


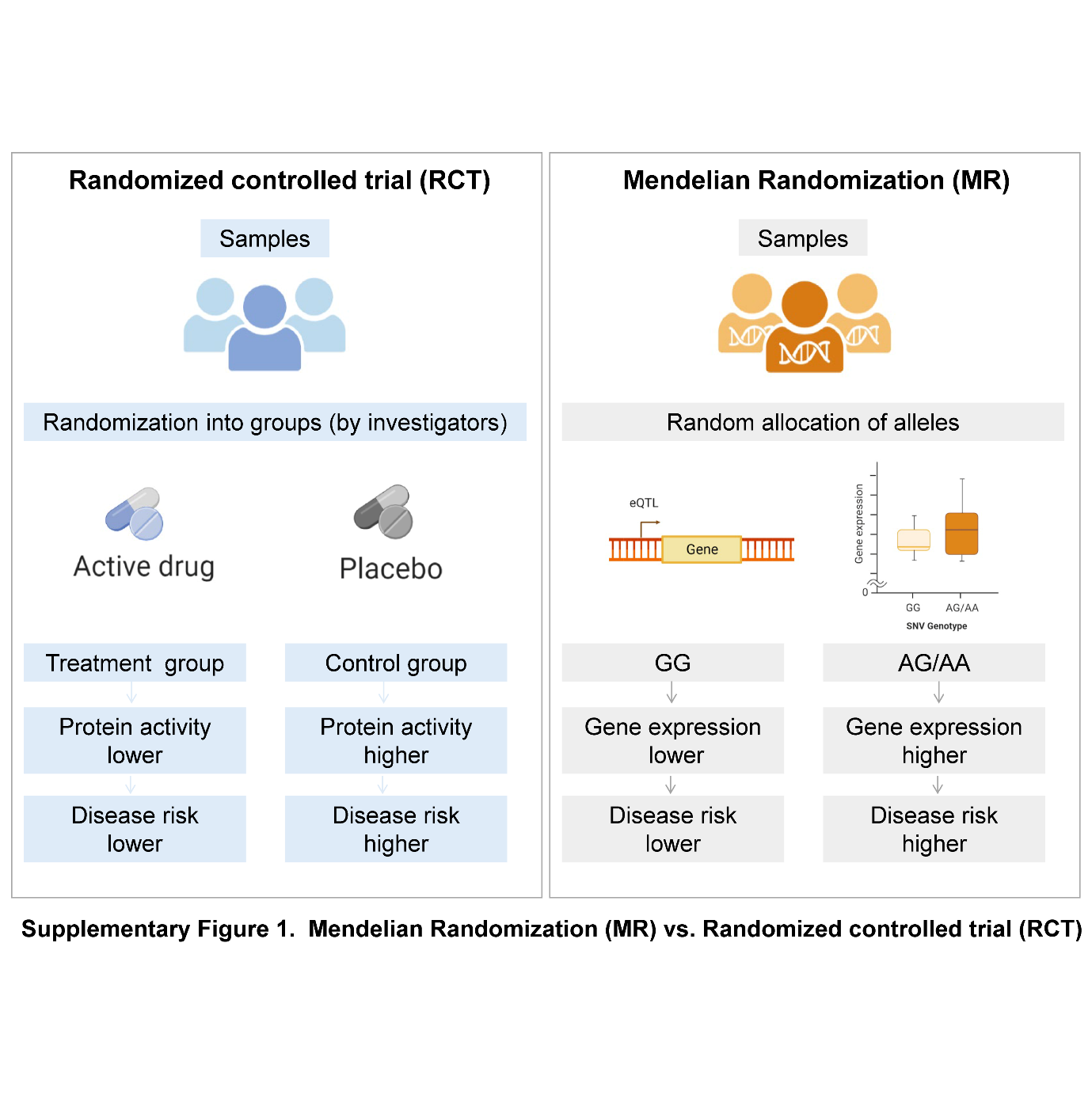


## Figure S2. Scatter plots of significant results from genetically predicted gene expression on MG risk in the primary analysis.


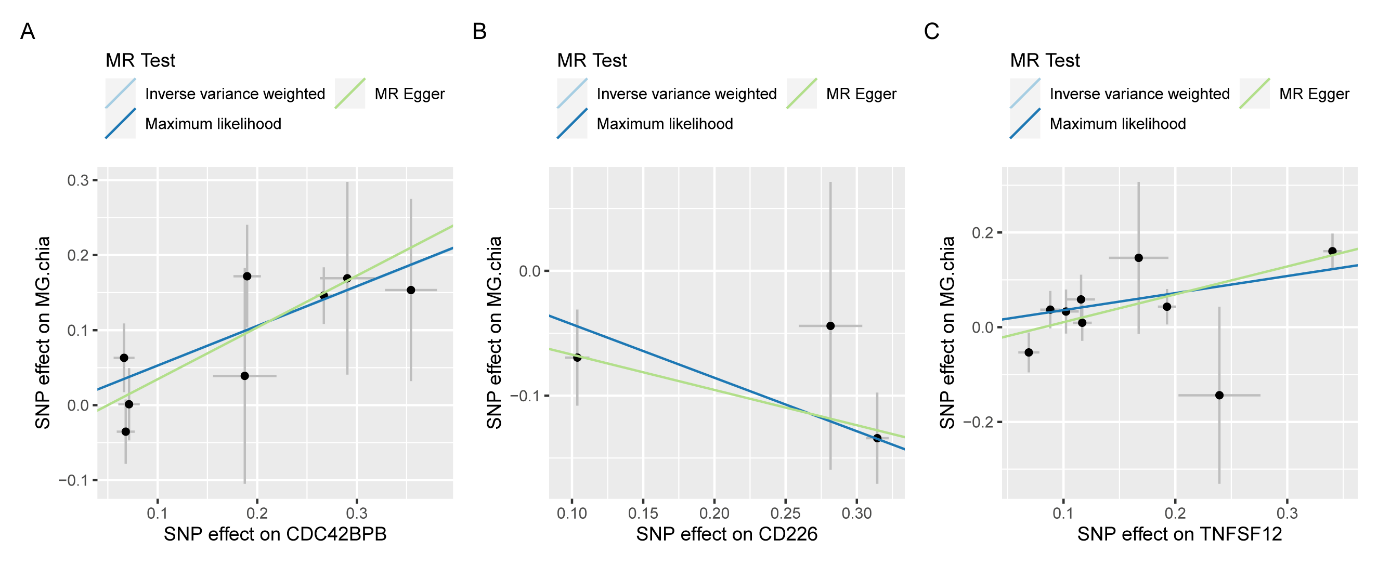


The outcome is MG GWAS from Chia et al. (1,873 patients and 36,370 controls)

## Figure S3. Forest plot showing MR estimate for genetically proxied gene and protein expression on MG outcome across different datasets.


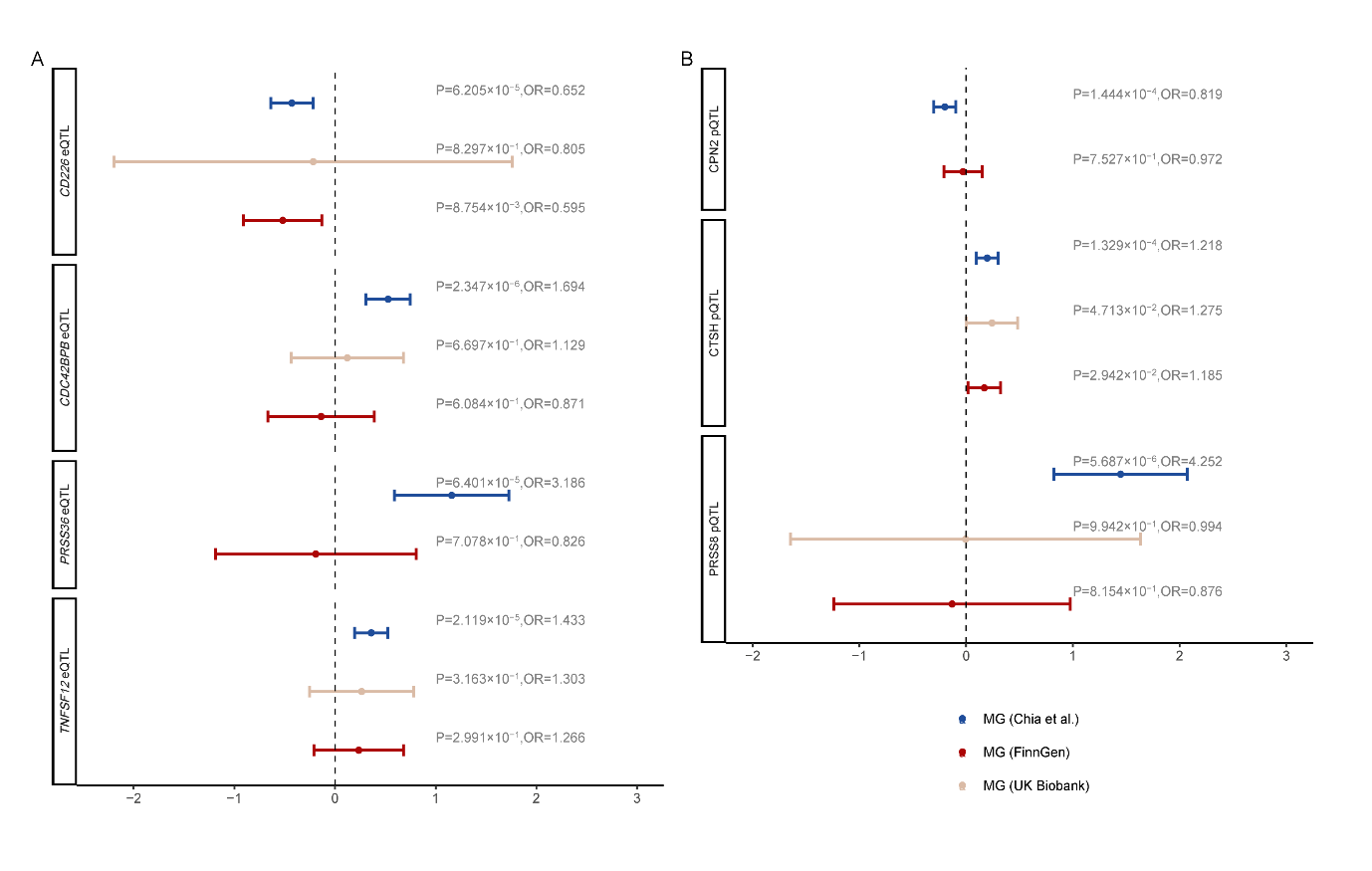


Forest plot showing MR estimate (95% CI) from two sample MR analyses. *P* are unadjusted. CI =confidence interval.

A. MR estimates of significant MR results used *cis-*eQTL instruments on MG outcome across different datasets.

B. MR estimates of significant MR results used *cis-*pQTL instruments on MG outcome across different datasets.

## Figure S4. PhenoScanner disease/trait annotation of the index eQTL/pQTL instrument with other traits.


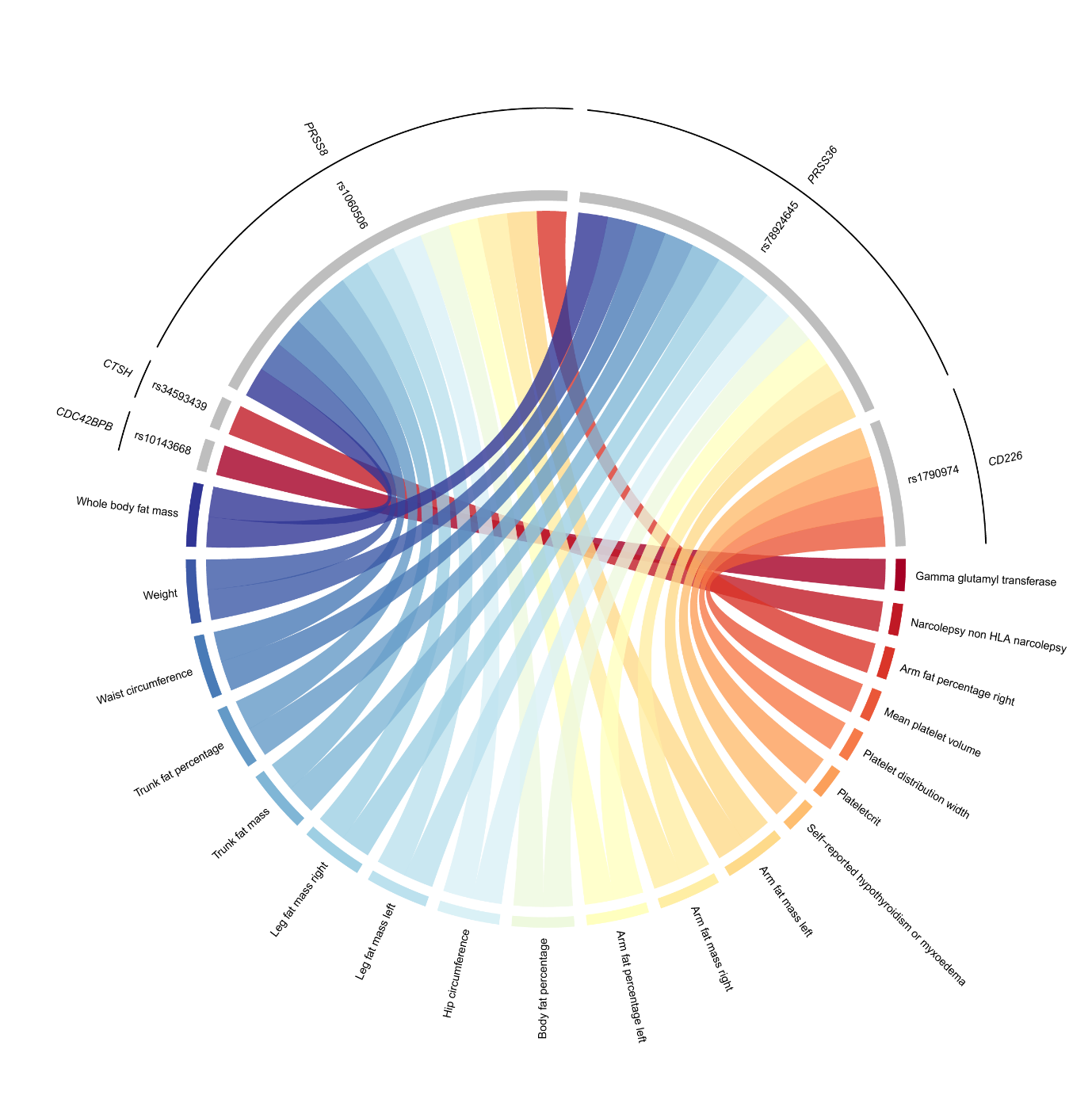


Only loci with a disease/trait annotation appear on this figure.

## Figure S5. LocusCompare plot depicting colocalization of the top SNP associated with eQTL surrounding CTSH in T_H_2 cell and MG GWAS.


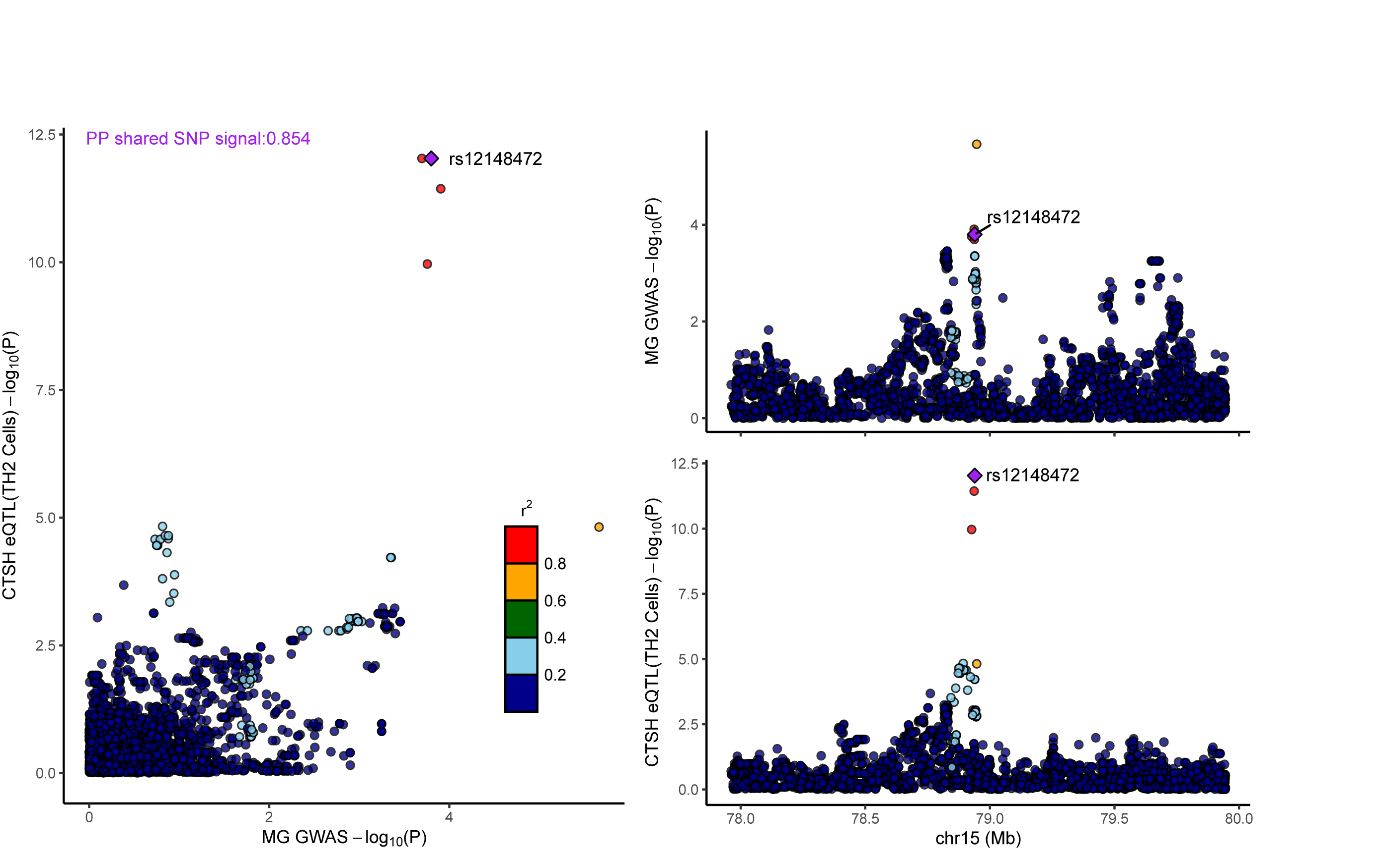


The top right plots show the association results in the MG GWAS; the bottom right plots represent the corresponding pQTL results; the left plot shows the colocalization of genetic association and eQTL signals. The SNP indicated by the purple diamond is the SNP for which the European LD information is shown.

## Figure S6. Protein-protein interaction of CPN2 using the STRING database (https://string-db.org/).


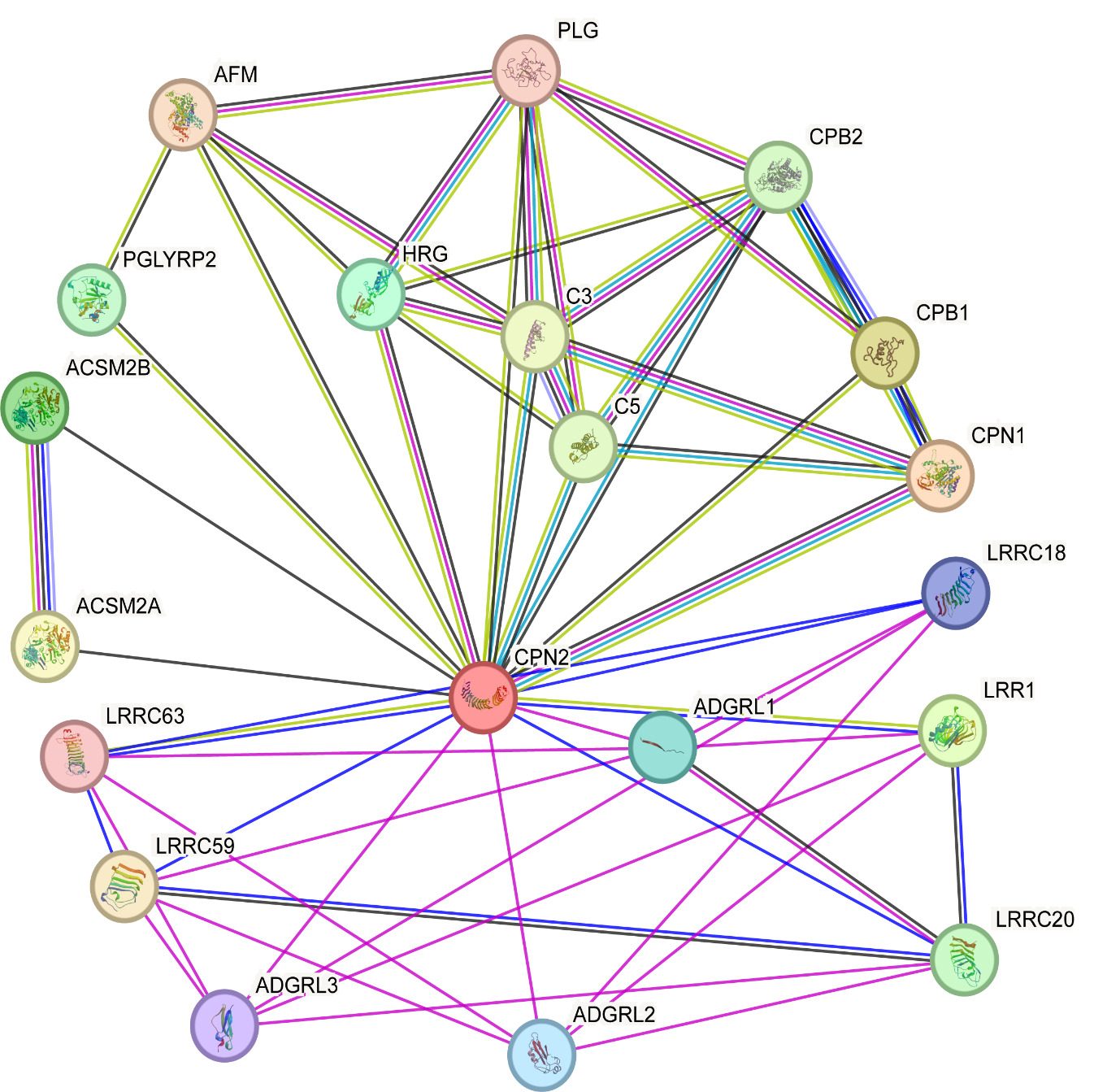


# Additional file 2

## Table S1. Basic Characteristics of eQTL Databases, pQTL Studies, and GWAS Datasets in the study.

## Table S2. An overview of druggable proteins and the coverage of genes/proteins in eQTLGen and pQTL studies.

## Table S3. Genome-wide significant loci in MG GWAS from Chia et al.

## Table S4. The *cis-*eQTLs instruments used for drug target gene expression on MG risk in the primary analysis.

The outcome is MG GWAS from Chia et al. (1,873 patients and 36,370 controls)

## Table S5. MR full results using *cis-*eQTLs on MG risk across different datasets.

## Table S6. Colocalization results using cis-eQTLs on MG across different datasets.

## Table S7. The *cis-*pQTLs instruments used for protein expression on MG risk in the primary analysis.

The outcome is MG GWAS from Chia et al. (1,873 patients and 36,370 controls)

## Table S8. MR full results of proteins using sentinel *cis-*pQTLs from six large proteomic studies on MG risk across different datasets.

## Table S9. Colocalization results using *cis-*pQTLs on MG risk across different datasets.

## Table S10. Colocalization results for genes/proteins using eQTL from the DICE database.
